# Vertigo

**Published:** 1920-06-19

**Authors:** 


### VERTIGO.

This term, in its psychological sense, suggests abandonment, a state of uncontrolled emotion, such as is so well demonstrated in that popular picture "La Vertige"; but in this article we must confine ourselves to a more limited conception of this word, and briefly discuss the relations existing between co-ordination, equilibrium, and orientation on the one hand, and in-co-ordination, instability, and dis-orientation on the other.

### Equilibrium in Motion.

Situated in the hinder part of the internal ear, in the petrous portion of the temporal bone, is an osseous labyrinth whose various passages constitute the semicircular canals. These canals are three in number, and lie in the three planes of space. Thus we have superior vertical, posterior vertical, and external horizontal. Each of these bony canals forms a closed tube, dilated at one end to form its ampulla, the lumen containing a fluid called perilymph, which does not entirely fill each canal, for, surrounded by the perilymph, is a close fibrous tube, the membranous semicircular canal, which also contains a fluid called endolymph. In this latter resides the local organ of equilibration, with its delicate hair-cells, whilst into the base of each cell the peripheral endings of the fibres of the vestibular nerve terminate, their central ends travelling into the brain and spinal cord.

When the perilymph is in any way disturbed, as by motion of the body, pressure changes are set up in it. These similarly affect the endolymph, any changes in which will instantly stimulate the hair-cells, and thus set up impulses in the peripheral endings of the vestibular nerves, whence impulses are interpreted and utilised by the central nervous system.

It is chiefly to the connections which they make with the cerebellar portion of the brain that nerves conveying messages to opposing destinations and for antagonistic functions are able to act in complete harmony. Thus the flexor muscles of a limb act against the extensors, and *vice versa*. It is necessary, therefore, that when performing flexion the corresponding extensors should receive no stimulus of action to contract or shorten, but rather actively to lengthen. Now, to bring this about requires some controlling centre where resides the master-key. This is the cerebellum and its subsidiary nerve centres in the cerebrum and spinal cord.

Psychical co-ordination is absolutely essential, not for movement only, but also if thought and its demonstration are to be reasonable. Concentration is the study of one picture, or set of pictures, to the temporary exclusion of all others. A brain thoroughly fatigued, or doped by drugs such as alcohol, loses the power of correct discrimination, so that confusion results, the wrong keys are turned, and antagonistic impulses set free simultaneously to wrangle in a maze of unreasonableness.

But to set forth the complete ætiology of vertigo may demand a careful and extensive diagnostic search, for, although but a small word, it represents a very wide area for investigation.

Medically, we may divide its causation into two classes—local and general; but it should be understood that the following classification must be necessarily incomplete.

### A. Local.

(1) Cerumen in the external auditory meatus in some cases. (2) Syringing the external auditory meatus and projecting fluid on to the tympanic membrane. (3) The various forms of acute and chronic disease directly affecting the deeper superficial parts of the internal ear—otitis media. Chronic destructive changes in the internal ear, due to tertiary syphilis, etc. Gun-concussion and shell-explosion, directly affecting the internal ear. Migraine.

### B. General.

Onset of many acute toxæmias; acute and chronic dyspepsia; certain cardiac lesions; arterio-sclerosis and high blood-pressure; certain destructive tumour and abscess lesions in the central nervous system; shell-shock—the general results of this condition lead to a severe form of neurasthenia, which itself may produce vertigo, apart from any aural lesion; severe emotion; influence of drugs, such as alcohol, tobacco (especially the first pipe or cigarette of the day), anæsthetics, etc.; asphyxia; climbing to a great height and looking down into space below; swinging to excess; rotation to excess; sea-sickness.

Many of these lesions are correlated to each other, but are mentioned separately for the sake of simplicity. There is a sensation as if the patient's immediate surroundings had decided to leave him, every object appearing to be in motion; he staggers, and may actually fall. Vertigo generally occurs suddenly and, except when due to serious organic lesions, is transitory. The patient who suffers from vertigo in its milder forms may become partially accustomed to such attacks, and to some extent come to disregard them, but always should he remember that such disease may be progressive, and that the cause must be investigated and removed if possible. For cases of true aural vertigo operation is sometimes called for, and frequently proves successful. Simple medical treatment can often give great relief when intelligently applied, and it cannot be urged too strongly that any person who suffers from recurrent vertigo, the cause of which is not obvious to himself, is well advised to submit to competent medical examination.

